# Alternative Surfactants for Improved Efficiency of In Situ Tryptic Proteolysis of Fingermarks

**DOI:** 10.1007/s13361-015-1140-z

**Published:** 2015-04-28

**Authors:** Ekta Patel, Malcolm R. Clench, Andy West, Peter S. Marshall, Nathan Marshall, Simona Francese

**Affiliations:** Biomolecular Research Centre, Sheffield Hallam University, Sheffield, S1 1WB UK; GlaxoSmithKline, Gunnels Wood Road, Stevenage, Hertfordshire SG1 2NY UK

**Keywords:** MALDI, In situ, Proteolysis, Detergents, Fingermarks

## Abstract

Despite recent improvements to in situ proteolysis strategies, a higher efficiency is still needed to increase both the number of peptides detected and the associated ion intensity, leading to a complete and reliable set of biomarkers for diagnostic or prognostic purposes. In the study presented here, an extract of a systematic study is illustrated investigating a range of surfactants assisting trypsin proteolytic activity. Method development was trialled on fingermarks; this specimen results from a transfer of sweat from an individual’s fingertip to a surface upon contact. As sweat carries a plethora of biomolecules, including peptides and proteins, fingermarks are, potentially, a very valuable specimen for non-invasive prognostic or diagnostic screening. A recent study has demonstrated the opportunity to quickly detect peptides and small proteins in fingermarks using Matrix Assisted Laser Desorption Ionization Mass Spectrometry Profiling (MALDI MSP). However, intact detection bears low sensitivity and does not allow species identification; therefore, a shotgun proteomic approach was employed involving in situ proteolysis. Data demonstrate that in fingermarks, further improvements to the existing method can be achieved using MEGA-8 as surfactant in higher percentages as well as combinations of different detergents. Also, for the first time, Rapigest SF, normally used in solution digestions, has been shown to successfully work also for in situ proteolysis.

**Electronic supplementary material:**

The online version of this article (doi:10.1007/s13361-015-1140-z) contains supplementary material, which is available to authorized users.

**Graphical Abstract:**
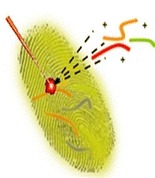
ᅟ

## Introduction

In Matrix Assisted Laser Desorption Ionization Mass Spectrometry Imaging (MALDI-MSI), the identification of observed proteins remains a challenge primarily due to the drop in sensitivity of time of flight (TOF) mass spectrometers beyond the mass range 25–30 kDa [1], inadequate mass resolving power at those molecular weights, as well as limited capabilities for top down approaches applied to singly charged ions and within samples with more than one protein. To counteract this, a “bottom up” approach is often employed; proteolysis yields smaller peptide fragments, typically between 500 and 3000 Da, which are easier to detect and with high mass accuracy. Whilst conventionally enzymatic digestion is carried out in solution (purified protein samples or from tissue homogenates), methodologies have been devised to digest proteins in situ; these protocols are applied to understand the function-localization relationship through preserving protein localization within a tissue. Though very informative, this strategy typically appears to yield a small number of identifiable peptides of low ion intensity when analyzed by mass spectrometry (MS); usually 20 at the most are identified by direct measurements [2]. This is very poor in comparison to conventional proteomics methodology (i.e., LC/ESI MSMS) applied to in-solution digests where several thousand peptides might be expected to be identified. This small number of identifiable peptides is in part due to the higher complexity and lower “extraction” yield of the peptide mixture obtained through the in situ digestions. The integration of ion mobility separation (IMS) within mass spectrometry analysis has shown to mitigate the complexity of the peptide mixture by resolving isobaric species, thus increasing and improving specificity and identification, respectively [3–5]. However, an important step in obtaining suitable and reliable protein signatures (including those from the less abundant proteins, which, in biomarker discovery and pathology diagnostics would have a game-changing effect), lies in improving the efficiency of in situ proteolysis.

The literature shows different ways for depositing the endopeptidase trypsin (the most efficient enzyme on tissue) for in situ proteolysis; sprayers produce a homogenous trypsin coating, which is necessary for successful imaging experiments of peptides and for their identification within their original locations [6, 7], whereas other methods, including robotic spotters [4, 8], have been reported to yield higher peptide signal intensity and a more abundant ion population. The advantages and disadvantages of these and other methods of application have been discussed in a recent review [9].

**Figure 1:**
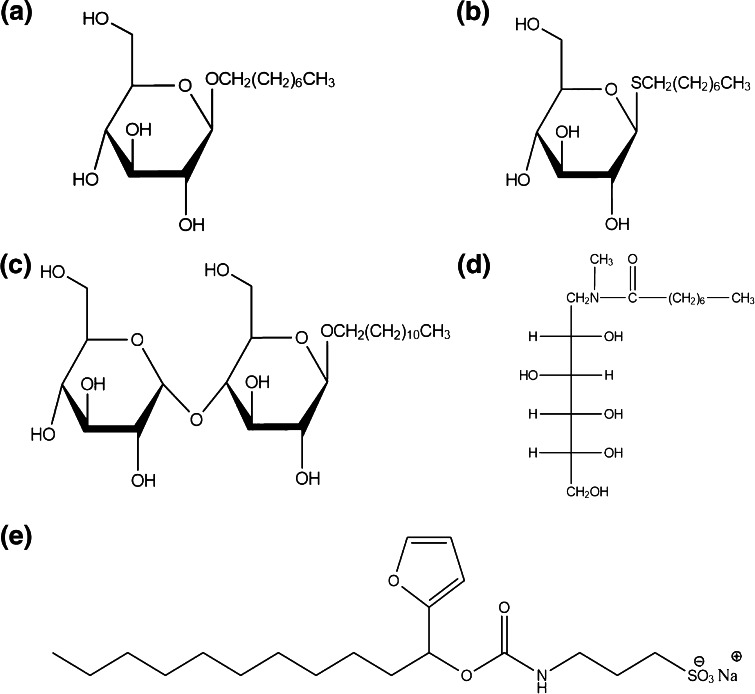
Chemical structures of non-ionic surfactants; **(a)** n-Octyl ß-D-glucopyranoside (OcGlu), **(b)** *N*-Octyl 1-thio-ß-D-glucopyranoside (OcThio), **(c)** *N*-Decyl ß-D-maltoside (DDM), **(d)** *N*-Octanoyl-*N*-methylglucamin (MEGA-8) and the anionic surfactant; **(e)** sodium 3-[(2-methyl-2-undecyl-1,3-dioxolan-4-yl)methoxyl]-1-propanesulfonate (RapiGest SF)

MS compatible detergents have been used to enhance in gel [10] and in solution [11] digestion of hydrophobic proteins, such as membrane proteins. Hydrophobic proteins can be proteolytically resistant to digestion because of inaccessible cleavage sites; therefore, in such instances, the limited number of peptides produced can ultimately affect protein identification. The amphiphilic nature of a detergent improves solubilization by unfolding the protein to make it more accessible to enzymatic cleavage, thus facilitating higher peptide coverage upon digestion. The incorporation of a detergent within in situ trypsin digest protocols has been previously described in a study of adenocarcinoma tissue sections to improve the yield of tryptic peptides [12]. Here, a systematic investigation of the use of a range of non-ionic and anionic surfactants, namely n-Octyl ß-D-glucopyranoside (OcGlu), n-Octyl 1-thio-ß-D-glucopyranoside (OcThio), n-Decyl ß-D-maltoside (DDM), and *N*-Octanoyl-*N*-methylglucamin (MEGA-8) (Figure 1a–d), (and combinations thereof) to further improve in situ enzymatic digestion is reported. Additionally the use of sodium 3-[(2-methyl-2-undecyl-1,3-dioxolan-4-yl)methoxyl]-1-propanesulfonate, which is marketed under the trade name of RapiGest SF (Waters Corporation, Manchester, UK), (Figure 1e), is reported for the first time in situ. This surfactant has been widely reported for its use in solution without interfering with MS analysis [13, 14] but surprisingly there are no reports of its use for in situ digests to date.

The initial investigations have been conducted on fingermarks. Fingermarks result from a transfer of sweat from an individual’s fingertip to a surface upon contact. As sweat contains, amongst many other classes of biomolecules, peptides and proteins, fingermarks can potentially represent a very valuable specimen to carry out non-invasive screening of biomarkers for prognostic or diagnostic purposes. A recent study has demonstrated the opportunity to quickly detect peptides and small proteins in fingermarks using Matrix Assisted Laser Desorption Ionization Mass Spectrometry Profiling (MALDI MSP) [15]. The detection of these species was exploited to determine the sex of an individual in a forensic context. However, some of these putatively identified species are indicated in the literature as possible biomarkers of conditions ranging from skin disorders [16] to cancer [17, 18]. Although the data reported by Ferguson et al. [15] are encouraging for the development of a future non-invasive screening methodology, fingermarks are a challenging specimen because of the low abundance of proteins in sweat (up to 15–25 mg/dL for eccrine sweat [19]).

While the addition of 0.1% OcGlu detergent was reported in earlier studies to greatly increase peptide yield [12], current investigations show that at increased concentrations, MEGA-8 is a more efficient detergent and many peptides and small proteins could be putatively identified exploiting the high mass accuracy of the instrument employed (SYNAPT G2 HDMS; Waters Corporation) operated in sensitivity mode. Furthermore, initial experiments have shown that RapiGest SF, a novel surfactant used in proteolysis carried out in solution, produces an increased number of peptide peaks in comparison to trypsin digestion alone, even when applied in on-tissue digestions.

These methods were applied, in initial studies, to the well characterized rat brain tissue in order to evaluate transferability of the protocols. Data from these investigations do overall demonstrate correspondence with the improved in situ proteolysis efficiency observed in fingermarks.

## Experimental

### Materials

α-Cyano-4-hydroxycinnamic acid (CHCA), aniline, acetonitrile (ACN), tri-fluoroacetic acid (TFA), ammonium bicarbonate, methanol (MeOH), ethanol (EtOH), chloroform (CHCl_3_), n-Octyl ß-D-glucopyranoside (OcGlu), *N*-Octyl 1-thio-ß-D-glucopyranoside (OcThio *N*-Decyl ß-D-maltoside (DDM), *N*-Octanoyl-*N*-methylglucamin (MEGA-8), phosphorus red and ALUGRAM SIL G/ UV254 pre-coated aluminium sheets were purchased from Sigma-Aldrich (Dorset, UK). RapiGest SF was purchased from Waters (Elstree, UK). Trypsin Gold, mass spectrometry grade (100 μg lyophilized) was purchased from Promega (Southampton, UK).

### Methods

#### Fingermark and Tissue Preparation

Ungroomed fingermarks were prepared and deposited onto aluminium sheets (which were pretreated to remove the stationary phase) as previously described [15]. Fresh frozen rat brain tissue sections were cut to 10 μm thickness using a Leica CM3050 cryostat (Leica Microsystems, Milton Keynes, UK) operating at –20°C. Sections were subsequently thaw-mounted onto poly-lysine coated glass slides and stored in an airtight tube at –80°C. All animal tissue studies were carried out in accordance with Animals (Scientific Procedures) Act 1986 and the GSK Policy on the Care, Welfare, and Treatment of Laboratory Animals.

#### In Situ Fingermark Digestion for MALDI MSP

Trypsin solution (20 μg/mL reconstituted in 50 mM ammonium bicarbonate, pH 8) containing either 10 mM OcGlu, OcThio, DDM, MEGA-8 or RapiGest SF, in concentrations varying from 0.5% to 2% w/v, was manually deposited (0.5 μL droplets) onto the fingermarks. The above trypsin solutions were also sprayed onto fingermarks (in order to evaluate data for future MS imaging experiments) using the SunCollect pneumatic sprayer (KR Analytical, Sandbach, UK); seven layers of trypsin were deposited at a flow rate of 1.5 μL/min and a nitrogen pressure of 3 bar. All samples were subsequently incubated in a parafilm-covered jar containing 50:50 H_2_O:MeOH for 3 h at 37°C (5% CO_2_). All digested fingermarks were subjected to MALDI MSP and peptide mass fingerprints were evaluated and compared.

Tissue sections were pre-treated with wash steps of 70% and 90% ethanol (1 min each) and chloroform (10 s); 20 μg/mL trypsin solution containing either 0.5% 10 mM OcGlu or 10 mM MEGA-8 was spotted (0.5 μL droplet) or sprayed (seven layers at a flow rate of 1.5 μL/min) using the SunCollect automated sprayer. Tissue sections were then incubated in a parafilmed jar for 4 h at 37°C (5% CO_2_).

A mixture of three detergents was also added to trypsin, 10 mM of each detergent: OcGlu, MEGA-8, and DDM were combined in the ratios of 1:1:1, 1:2:1, and 1:1:2, and 0.5% w/v of this detergent mixture was added to 20 μg/mL trypsin solution.

#### Matrix Deposition

Ten mg/mL CHCA in 50:50 ACN:0.5% TFA_aq_ containing equimolar amounts of aniline to CHCA (i.e., one mL of 10 mg/mL CHCA solution contained 4.8 μL aniline) was spotted using an automatic pipette (0.5 μL droplet) onto the localized digest regions. Five layers of 5 mg/mL CHCA (same composition) were sprayed using the SunCollect autosprayer (KR Analytical) at a flow rate of 1.5 μL/min onto those samples that were previously sprayed with trypsin solution.

#### Instrumentation and Data Acquisition

Calibration over a 600–2800 Da mass range was performed prior to analysis using phosphorous red. MALDI IMS/MS data and MS images were acquired in positive ion mode from 600 to 2800 Da at a mass resolution of 10,000 FWHM using a SYNAPT G2 HDMS system (Waters Corporation) operating with a 1 KHz Nd:YAG laser. Three full scan mass spectra were manually acquired over 30 s; all experiments were carried out in triplicate. The laser energy was adjusted as per sample type between 200 and 220 arbitrary units on the instrument. Image acquisition was performed at 100 μm spatial resolution in positive mode.

#### Data Analysis

Mass spectra obtained from MassLynx (Waters Corporation) were either converted into .txt files and imported into mMass [20, 21], an open source multiplatform mass spectrometry software, or processed directly within MassLynx by means of peak smoothing, baseline correction, and peak centroiding. MSI data were processed using the high definition imaging (HDI) software (Waters Corporation). Expasy (http://www.expasy.org/) was employed to generate in silico peptide lists of known proteins present in fingermarks (from examining the literature available) and rat brain. In silico peptide lists were generated by selecting “*human*” as taxonomy for fingermark analysis and “*rat*” for the brain tissue investigated. Mass lists were generated by selecting “*monoisotopic,*” “*MH*^*+*^,” “*trypsin higher specificity,*” “*2 missed cleavages,*” and “*methionine oxidation.*” *P*eptide lists were imported into mMass to create an “in house” and local reference library. Mass lists including known matrix (or matrix cluster, adduct) and trypsin autolysis *m/z* were used to preliminarily assign peaks and, therefore, exclude them from subsequent peptide assignment. Peak assignments in mMass were performed automatically using the “*compound search*” tool and the created library by setting the tolerance at 10 ppm with a “*max charge*” of 1 and ticking the box “*monoisotopic.*” Prior to peak assignment search, spectra were smoothed and de-isotoped. Peak assignment was not accepted if the S/N was lower than 3:1; peaks were not considered as peptides if their *m/z* it did not fall within the 0.4–0.8 fractional range.

To identify significant differences in the performance of the detergents, StatsDirect statistical software (ver. 2.7.8) was used. Data were shown to be nonparametric via Shapiro Wilk test; therefore, a Kruskall-Wallis with Conover-Inman post hoc analysis test was implemented (*P* ≤ 0.05).

## Results and Discussion

**Figure 2:**
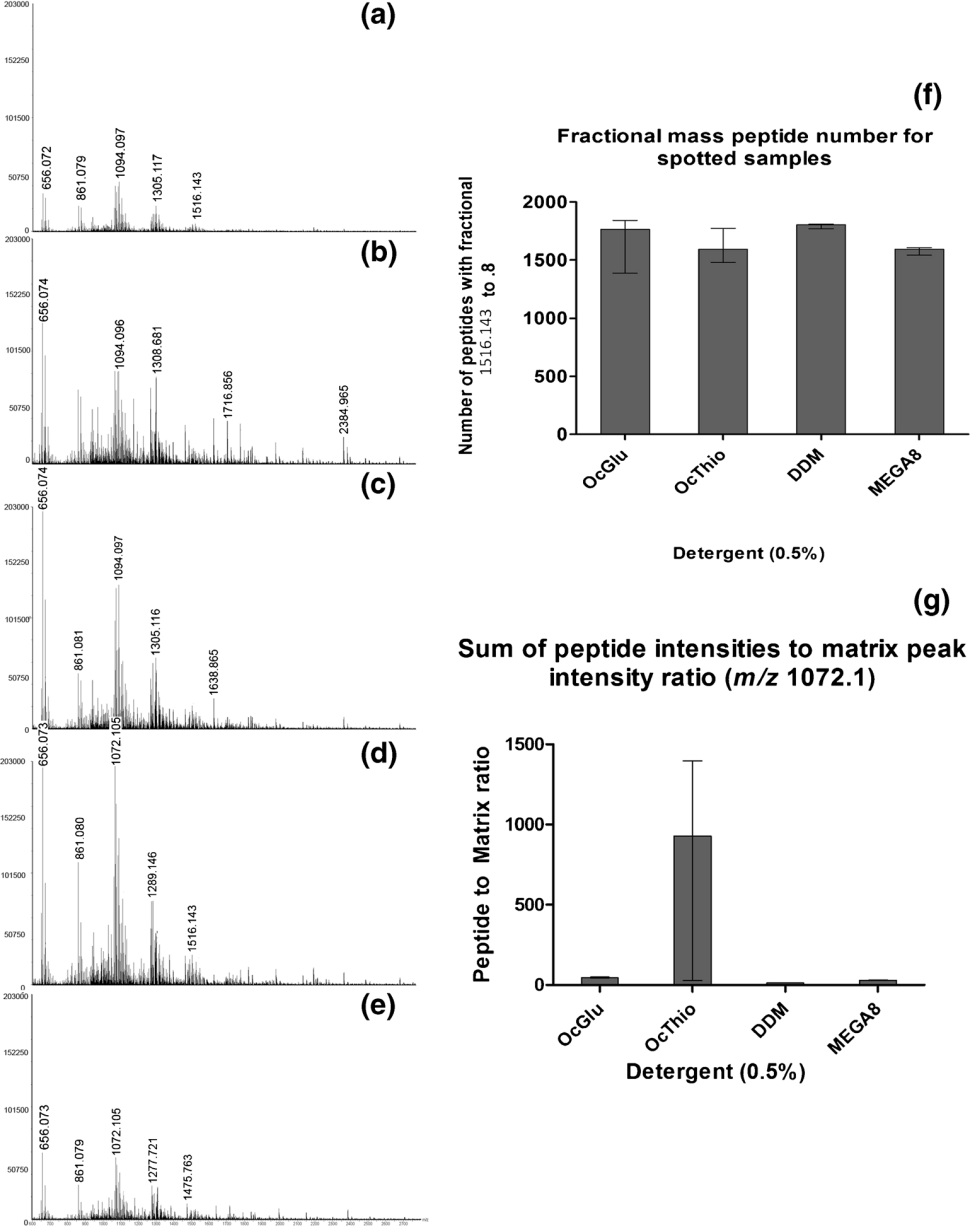
MALDI MS peptide profiles from in situ digests of ungroomed fingermarks spotted with 20 μg/mL trypsin alone in 50 mM ammonium bicarbonate pH 8.04 **(a)**, or containing 0.5% concentration of the detergents **(b)** OcGlu, **(c)** OcThio, **(d)** DDM, or **(e)** MEGA-8. Column graph showing the number of peptides with fractional mass between 0.4 and 0.8 **(f)** and corresponding peptide:matrix intensity ratio for each detergent **(g)**. The consistently detected matrix peak at *m/z* 1072.1047 ([CHCA – 4H + 4Na + 1 K]^+^) was selected for the calculation of this ratio

This study aimed to investigate the efficiency of a range of potentially useful detergents to increase the ion abundance and ion population from fingermark protein-originating peptides, within a shotgun proteomic approach involving in situ proteolysis prior to MALDI MS analysis. In the initial stage of method development, proteolysis was performed with and without the presence of surfactants. In the former case, trypsin solutions containing 0.5% of either of the non-ionic detergents, OcGlu (conventionally used detergent since the work of Djidja et al. [12]), OcThio, DDM, and MEGA-8, were spotted onto ungroomed fingermarks. Mass spectra generated from the direct profiling of digested fingermarks are displayed in Figure 2, showing that the addition of any of the detergents used results in higher ion abundance and ion population, compared with trypsin alone (Figure 2a), in agreement with previous observations for on-tissue digests. Generally, OcGlu and OcThio exhibit similar peptide peaks, albeit in higher intensities within the OcGlu digest (Figure 2b and c, respectively) and this could be expected because of the near-identical chemical structures (Figure 1a and b).

From manual spectral interrogation, OcGlu and OcThio appear to yield a greater number of tryptic peptides in the lower mass region, whereas DDM (Figure 2d) shows a richer peptide ion population throughout the mass range acquired. For a more objective and systematic evaluation, the majority of the in silico digests of the known protein targets reported were examined; it was observed that apart from exceptions (i.e., *m/z* 2038.0161 QEIECQNQEYSLLLSIK from Keratin-9), the *m/z* of peptides fall within the fractional range 0.4–0.8 (in line with what the proteomic community considers the fractional range of peptides to fall within). Therefore, only the *m/z* signals falling within this range were considered as peptides in the spectra; these peptide signals were used (their number and relative intensity) to allow a uniform relative comparison between the performance of the different detergents. Figure 2f shows the median values with a range for the number of peptides between 0.4 and 0.8 for each detergent. All the detergents yielded a comparable number of peptides (Figure 2f). OcGlu, followed by OcThio, produced the largest range comparing data from the three repeat experiments; conversely, the small range on the DDM and MEGA-8 bars demonstrated reproducibility.

The MALDI mass spectrum generated by employing MEGA-8 as a detergent has been used to search for proteins that have been shown to be present in fingermarks or sweat by others using different non-in situ approaches [19, 22]. As the mass accuracy of the instrument upon calibration was 10 ppm, putative assignments, reported in Table 1, were made on the basis of the peptide maps using in silico digestions using the bioinformatic tools hosted by the Expasy portal (http://www.expasy.org/). Interestingly, psoriasin, an antimicrobial protein, putatively detected by Ferguson et al. [15] in the intact analysis, was also detected here through a peptide at nominal *m/z* 2750.2882 with a relative error of 9 ppm. Taking stock from these observations, further method development involved in situ digestions performed by spraying the trypsin solution instead of spotting it, with the view to enable MALDI MS imaging to be applied to fingermark specimens as well as to other tissues in the future. Given their performance in the spotting experiments, OcGlu, DDM, and MEGA-8 were employed in two separate experiments: (1) application of the trypsin solution containing either of these three detergents through spray-coat and (2) spray coat application of a trypsin solution containing all of the three detergents in order to maximize coverage and encompass the benefits of each detergent from the spotting experiments. OcGlu has been published as a proven additive to enhance proteolytic digests [7, 12]; although current investigations show that this detergent produced a large range of peptide numbers across the three repeat experiments (Figure 2f), the inclusion of this detergent was used for comparative purposes.

**Table 1:** Peptide Mass Fingerprinting and Putative Protein Identifications from In Situ Fingermark Digests Performed by Spotting a Trypsin Solution Containing 0.5% MEGA-8 as a Detergent

PROTEIN	PEPTIDE *m/z*	SEQUENCE	RELATIVE ERROR (PPM)
Psoriasin	2750.2882	ENFPNFLSACDKKGTNYLADVFEK	–9.0
Keratin type I	2187.0196	SDLEMQYETLQEELMALK [Met Ox]	0
Human serum albumin	1898.9799	RHPYFYAPELLFFAK	–8.0
	2346.9887	TYETTLEKCCAAADPHECYAK	–6.5
Alpha-2-glycoprotein 1 zinc	2035.0434	IDVHWTRAGEVQEPELR	3.9
Aspartate aminotransferase mitochondrial	2216.9593	NLFAFFDMAYQGFASGDGDK [Met Ox]	1.96
Calmodulin-like protein 3	2117.0760	ELGTVMRSLGQNPTEAELR [Met Ox]	5.0
Corenodesmosin	2750.2882	SIGTFSDPCKDPTRITSPNDPCLTGK	–8.8
Filamin B	2064.0684	SPFEVSVDKAQGDASKVTAK	3.7
Kallikrein-11	2102.9956	GFECKPHSQPWQAALFEK	–7.6
	2403.0369	CENAYPGNITDTMVCASVQEGGK [Met Ox]	

In order to determine whether the overall intensity of peptides changes across the spectra generated by the different detergents, the ratio between the sum of peptide intensities against a single matrix peak intensity (*m/z* 1072.1047, [CHCA – 4H + 4Na + 1 K]^+^) was calculated (Figure 2g). Taking into account three repeats, OcGlu produced spectra with the highest peptide:matrix intensity ratio (average of 46.5:1); MEGA-8 consistently produced spectra containing peptides of an overall higher relative intensity (average of 29.8:1) than DDM-generated peptides (average of 12.7:1 respectively). OcThio was not carried forward into the second set of experiments as, in terms of peptide:matrix intensity ratio in the spotting experiments, the range of values across the three mass spectra replicates was much higher than that yielded by OcGlu (that is, the use of OcThio gives rise to hot spots) (Figure 2g).

**Figure 3:**
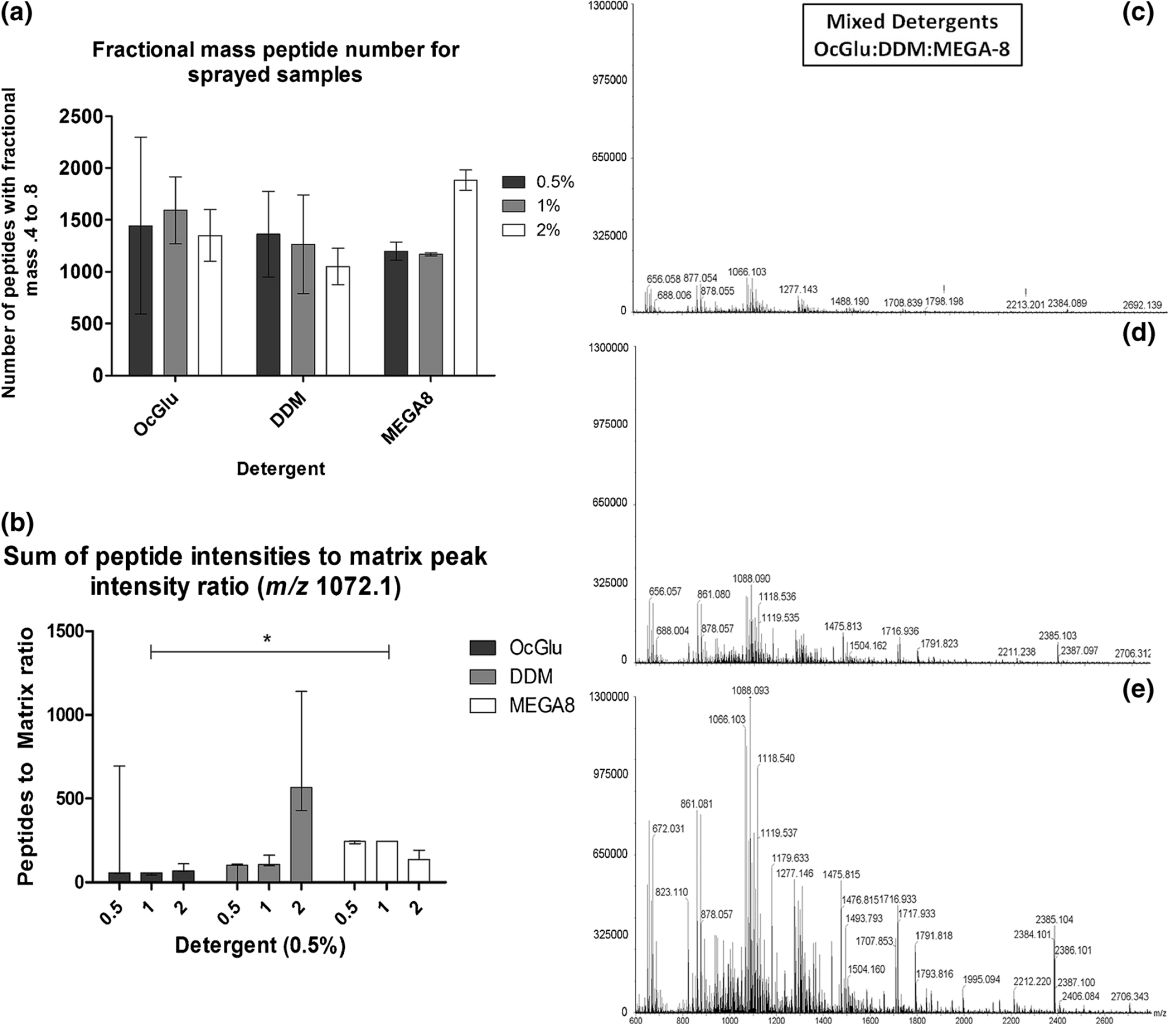
Column graph of the number of peptides with fractional mass between 0.4 and 0.8 for OcGlu, DDM, and MEGA-8 at three concentrations **(a)**. Graphical representation of peptide intensity:matrix intensity ratios for each detergent at three concentrations. Statistical analysis calculated a significant increase between MEGA-8 and OcGlu at 1%, as denoted by the asterisk **(b)**. Additionally, representative peptide profiles from the three detergents when mixed together 0.5% concentration (OcGlu:DDM:MEGA-8) are reported for various relative ratios of **(c)** 1:1:1, **(d)** 1:2:1, and **(e)** 1:1:2

In experiment (1), on-tissue digests by trypsin spray-coating were carried out on ungroomed fingermarks using either 0.5%, 1%, or 2% detergent. Figure 3a shows the number of peptides with a fractional mass between 0.4 and 0.8 represented as median values with range. Of the three detergents, MEGA-8 produced reproducible peptide numbers across the three experimental repeats as demonstrated by the narrow ranges. It is apparent that the 2% MEGA-8 digest produces the highest number of peptides (Figure 3a), which corroborates the detailed visual inspection of the mass spectral data when zooming in the spectrum (data not shown). The number of peptides varied across replicates for both OcGlu and DDM at all three concentrations, as indicated by the ranges. Interestingly, for all of the three detergents at 0.5% concentration, generally the peptide intensity:matrix intensity ratio is actually higher when the trypsin solution is sprayed compared with when it is spotted. This could be due to the low abundance of proteins in this particular specimen investigated (lower than in classic tissue sections) and simply, when spraying, the ratio detergent/endogenous proteins and/or trypsin/endogenous proteins, could be more favorable than those from spotting experiments. For method development, this is an encouraging observation towards future imaging experiments as it shows that signal intensity is not compromised when trypsin is sprayed. Second panel on the right hand side of Figure 3 shows representative peptide profiles from the three detergents when mixed together and added to 20 μg/mL trypsin solution at 0.5% concentration (OcGlu:DDM:MEGA-8) at various relative ratios of (c) 1:1:1, (d) 1:2:1, and (e) 1:1:2. Trypsin containing *at any one time* the three detergents at 1% yielded very similar distribution of peptides to those obtained using the detergents in 0.5% concentration (Supplementary Figure S1D–F). However, notably, all three detergents at 1% concentration yielded overall much lower intensities for the same peptides. In theory, especially from the 1% detergent concentration experiments, it could be expected that an even higher concentration of detergent may result in lower trypsin efficiency because of possible denaturation of the enzyme or peptide ion suppression. However, this was not the case for DDM or MEGA-8. Remarkably, using 2% MEGA-8, MALDI spectra contain both the highest number of peptides and peptide signals exhibiting the highest intensity overall (Figure 3a and b). From conducting this systematic study it is apparent that it is reasonable to measure the efficiency of a detergent based on three criteria: the ability to (a) yield the highest number of peptides, (b) yield the highest peptide:matrix intensity ratio overall, and (c) to reproducibly yield (a) and (b) across three replicates.

Whilst DDM at 2% produced the highest peptide:matrix intensity ratio (Figure 3b), the large range across replicates and considerably low number of peptides produced in comparison to MEGA-8 at 2% (Figure 3a) would indicate that overall MEGA-8 was the more efficient detergent. In contrast to the spotting experiments, these spectra allowed peptide mass fingerprinting to be undertaken by assigning multiple peptides for many of the tentatively assigned proteins. For example, keratin type 1, previously putatively identified by assigning one peptide, through spotting a trypsin solution containing 2% MEGA-8, is now identified through two peptides at lower *m/z* (*m/z* 1060.5605 and 1323.6727) in the spraying experiments. Interestingly, dermcidin, a small antimicrobial protein, putatively detected by Ferguson et al. [15], was also detected here through five peptide assignments at *m/z* 676.3829, 725.3932, 1128.5365, 1459.7622, 1466.7872. Here it is important to actually specify that this assignment could be made to dermcidin or a dermcidin-derived species originating from *the in vivo* cleavage of this small protein. However, at this stage, this accurate information, possibly requiring the use of a second enzyme to confirm, is outside the scope of this paper. Another antimicrobial species, LL-37, the C-terminal part of the only human cathelicidin identified to date called human cationic antimicrobial protein (hCAP), was also putatively detected through the peptide at *m/z* 1365.6610. The peptide assignments for this “spray-coat” 2% MEGA-8 experiment are shown in Table 2. The spraying method holds the promise to be able to identify lower abundance species since both dermcidin and LL-37, absent in the spotting experiments, are present in sweat at a lower abundance than serum albumin and keratin (previously identified in the spotting experiments). Also, it was observed that in the spraying experiments, protein identifying peptides were generally of lower *m/z* than those in spotting experiments; just as one example, psoriasin was putatively identified through the peptide at *m/z* 2750.2882 in the spotting experiments and through the peptide at *m/z* 1385.7194 in spraying experiments. We speculate that this is due to a higher efficiency of analyte extraction in the spotting experiments, especially with regards to higher *m/z* species. However, identifications through lower *m/z* peptide maps are advantageous as these species would be easier to fragment and have generally higher signal intensities. It should be taken into account that the overall proteolysis efficiency (number of peptides generated and intensities) is not solely dependent on the intrinsic efficiency of the detergents but also on the nature and abundance of the proteins [8]. As different detergents may denature specific classes of proteins, it can be hypothesized that mixing the detergents in appropriate ratios could allow the benefits of each to be combined to increase the number and types of peptides and proteins identified [11].Therefore, detergents were mixed and tested in experiment (2) at different relative ratios: (a) OcGlu:DDM:MEGA-8 at 1:1:1, (b) 1:2:1, and (c) 1:1:2. The surfactants were used at different concentrations and, in the mixed detergents experiments, the 0.5% concentration worked best (data not shown). When examining the resulting MALDI mass spectra, in Supplementary Figure S1A–I, the ion population appears to be confined to <*m/z* 1800 for the individual detergents, whereas the spectra originating from using the mixed detergent “formulation” produces an even spread of signals from *m/z* 600 through to *m/z* 2800 (Figure 3c–e), thus verifying the initial hypothesis. As anticipated, the benefits encompassed from the three detergents can be seen with ion population spread across the entire mass range regardless of the differences in detergent proportions, though, amongst the trialled ratios, the 1:1:2 ratio yielded the highest peak intensity and the richest ion population. Though the entire mass range was more ion populated, the ion intensity was generally lower than that observed for the application of the individual detergents. Therefore, to further improve the peptide intensities, future work should be carried out testing the same ratio albeit with a higher surfactant concentration (from 0.5% to 2%), in particular of MEGA-8, given that this detergent showed to be working at its best when present in 2% concentration.

**Table 2:** Peptide Mass Fingerprinting and Putative Protein Identifications from In Situ Fingermark Digests Performed by Spraying a Trypsin Solution Containing 2% MEGA-8 as a detergent

PROTEIN	PEPTIDE *m/z*	SEQUENCE	RELATIVE ERROR (PPM)
Psoriasin	1384.7194	KGTNYLADVFEK	6.2
Keratin type I	1060.5605	TLLDIDNTR	–2.6
	1323.6725	IKFEMEQNLR [Met Ox]	0
Keratin 1B	967.4723	DVDAAYVSK	–0.8
Antibacterial protein LL-37 134-170	1365.6610	WALSRGKR	–8.5
Adrenomedullin	1060.5605	SIGTFSDPCKDPTRITSPNDPCLTGK	–7.7
Beta-defensin 103 precursor	703.3621	EEQIGK	4.8
	933.4896	CAVLSCLPK	–2.3
Dermicidin	676.3829	SSLLEK	–6.9
	725.3932	GAVHDVK	–1.1
	1128.5365	ENAGEDPGLAR	7.6
	1459.7622	LGKDAVEDLESVGK	–1.0
	1466.7872	GAVHDVKDVLDSVL	1.6

**Figure 4:**
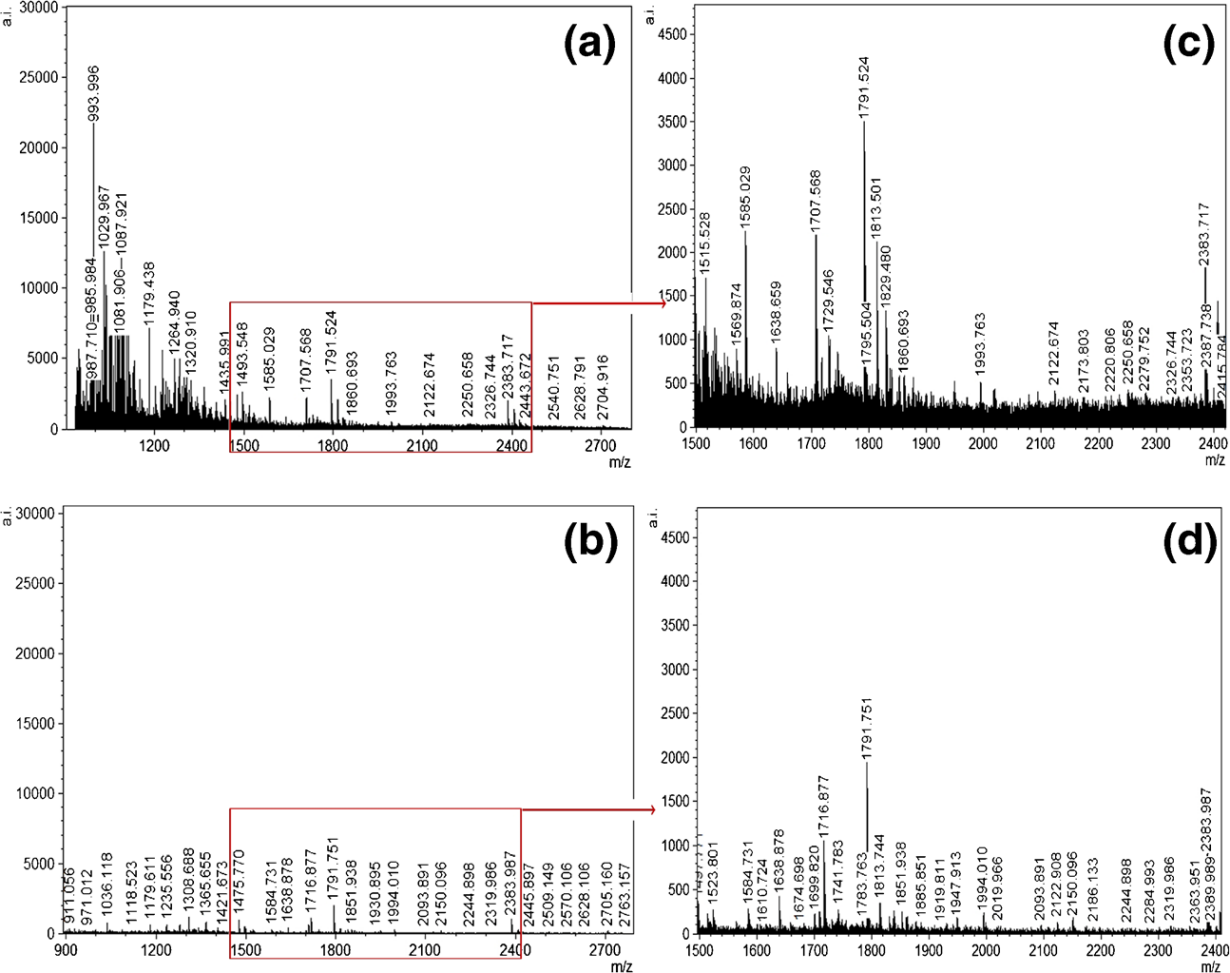
MALDI MS spectra of ungroomed fingermarks digested with and without RapiGest SF. The proteolytic solution was applied by spray-coat. **(a)** MALDI MS spectrum generated using a 20 μg/mL trypsin solution containing. RapiGest SF at 0.1% concentration; **(b)** MALDI MS spectrum generated using a 20 μg/mL trypsin solution with no RapiGest SF. Panels **(c)** and **(d)** display a zoom in the regions between 1500-2400 *m*/*z* for the spectra in panels **(a)** and **(b)** respectively

Another surfactant of interest in these investigations was RapiGest SF. MALDI MS spectra resulting from the addition of RapiGest SF to trypsin sprayed onto an ungroomed fingermark shows an improvement in the number of tryptic peptides produced and increase in ion intensity (Figure 4a) compared against trypsin alone (Figure 4b). In particular, there are peptide peaks between *m/z* 1000 and 2000 Da, present in the RapiGest SF generated peptide profile that are absent in the tryptic digest alone. RapiGest SF was used at 0.1%, which is the optimum concentration suggested by the manufacturer Waters for in solution experiments. Additionally, results indicate that the inclusion of 0.1% RapiGest SF produces a similar number of ion signals as for the 2% MEGA-8 digests, albeit of lower intensity, except in the 1000–1200 Da mass region, showing good scope for further experiments for the optimization of the RapiGest SF concentration. However, the novel in situ use of RapiGest SF reported in this study may require further development in order to determine the optimal concentration for this kind of proteolysis.

In view of making this alternative protocol useful for other biological specimens, additional experiments were carried out on fresh frozen rat brain tissue comparing the performances of sprayed OcGlu and MEGA-8 detergents (the most efficient detergents) at 0.5% concentration. The spectra generated several hundred peaks with S/N ratio above 3.5. Most of the peaks were considered to be from the most abundant proteins such as the putatively identified myelin basic protein (MBP); these ion signals were assigned using a 10 ppm mass tolerance and included *m/z* 615.2892 (DSHTR, 7.6 ppm error), 643.3698 (RPSQR, 9.9 ppm error), 705.3278 (SGSPMAR, 9.9 ppm error), 709.3462 (FSWGGR 6.4 ppm, MBP isoform 4), 726.4108 (HGFLPR, 8.7 ppm error), 811.4387 (TPPPSQGK, 9.7 ppm error) and 1339.7151 (HRDTGILDSIGR, 5.6 ppm error) which acted as a reference for evaluation of the different detergent performances. This comparative strategy, was informed by the work of the European Union COST Action Programme *BM1104 "Mass Spectrometry Imaging: New Tools for Healthcare Research"* in which the authors are participants. This program has networked 24 countries having, amongst others, excellent expertise in in situ tryptic proteolysis; after carefully designing a round robin study (agreed by the 24 countries), MBP (an extremely abundant protein in brain tissue) was selected as a target to compare trypsin digestion conditions for rat brain tissue and draw conclusions on best practice and most efficient protocols. The paper is in preparation (under revision of the co-authors) and will be soon submitted.

One additional finding was that under closer examination of the higher mass regions, peak intensities originating from the use of MEGA-8 are clearly superior to those of OcGlu as shown in Supplementary Figure S2, in agreement with the corresponding observations for fingermark digests. Also, less peptides could be putatively assigned to MBP when using 0.5% OcGlu in the proteolytic solution (*m/z* 615.2835, 643.3671, 709.3480, 1460.7118).

**Figure 5:**
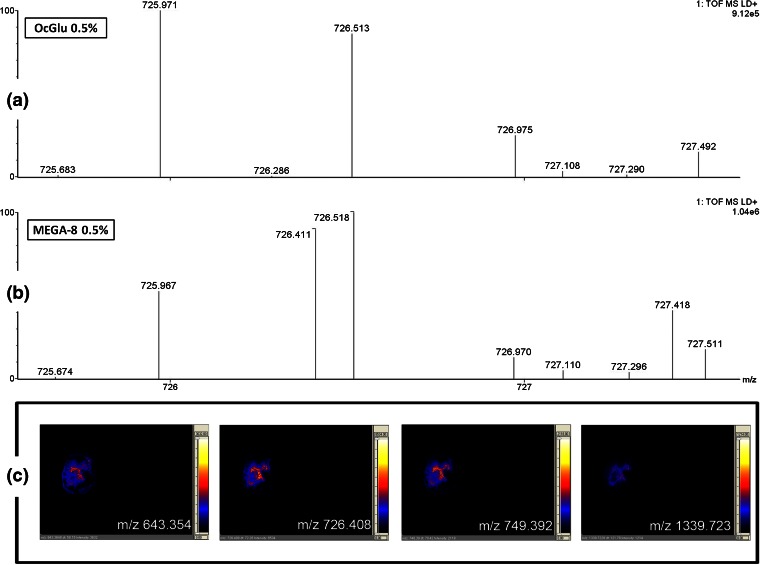
Comparative evaluation of OcGlu and MEGA-8 performances for the in situ digestion of rat brain sections. MALDI MS spectra is shown (in a very narrow *m/z* region) of the in situ digestion using 20 μg/mL trypsin solution containing **(a)** OcGlu at 0.5% concentration and **(b)** MEGA-8 at 0.5%, to show the presence of the MBP peptide at *m/z* 726.4 in the MEGA-8 digest and its absence in the OcGlu digest. Indeed, many MBP signals were absent in the spectrum when using OcGlu as a detergent instead of MEGA-8 with lower intensity peptide peaks; **(c)** demonstrates the opportunity for chemical mapping through MALDI MSI using 0.5% MEGA-8 as a detergent. Many peptide signals could be mapped, including those for MBP at *m/z* 643, 726, 749, and 1339; the latter images show identical distribution on the section, thus supporting the ion assignments to MBP

In Figure 5, a representative example of the above observations is reported in which the peptide at *m/z* 726.4108 is absent in the 0.5% OcGlu spectrum digest (Figure 5a), is instead clearly present at high intensity within the 0.5% MEGA-8 spectrum digest (Figure 5b). A rat brain tissue section was also submitted to MALDI MS imaging using 0.5% MEGA-8 in the proteolytic solution showing the opportunity for efficient chemical mapping (Figure 5c).

The mixed detergent trypsin solution previously trialled using fingermarks was also employed to digest rat brain tissue using a 0.5% concentration for each detergent at the same ratios previously tested. This time OcGlu:DDM:MEGA-8 1:1:1, and not the 1:1:2 ratio, produced the best peptide profile of the three mixed detergent digestion (Supplementary Figure S3), and tentative identifications were made as to the presence of MBP peptides (*m/z* 726.328 and 1339.556). However, in this case the mixed detergent tryptic solution produced an overall lower spectral intensity than OcGlu and MEGA-8 applied separately. This suggests that it cannot be assumed that one protocol is directly transferable from one sample type to another, and protocol refinements are indeed needed when developing a mixed detergent method for a different tissue model. Finally, rat brain tissue was digested with trypsin containing 0.1% RapiGest SF to evaluate transferability of this novel application method for this detergent to other specimen. The resulting mass spectrum exhibited peptides of high intensities in the low to mid mass range (*m/z* 600–1500), as shown in Supplementary Figure S4. Peptide peaks were generally of higher intensity compared with those generated from the mixed detergents proteolytic solution, though, as observed above, further optimization is needed both for the RapiGest SF and the mixed detergents when applied to rat brain tissue in this case.

## Conclusions

Despite improvements to on-tissue digestion efficiency, the quest remains for methods to further improve both reliability and number of the proteins identified when using a bottom up proteomic approach. The study illustrated in this paper embarked on a systematic study to discover possible alternatives to the use of OcGlu for even more improved on-tissue proteolysis. For initial developments, fingermarks were employed as a challenging substrate and also in view of using this specimen in the future as a means on noninvasive disease screening. The study concluded that concentrations of OcGlu higher than 0.1% (up to 2%) are compatible with MALDI-MS based analysis and that although this detergent is the most popular one for efficient proteolysis, others could be employed with even higher efficiencies and reproducibility such as the non-ionic surfactant MEGA-8. Indeed, this detergent was shown to work better at 2% concentration, especially when the proteolytic solution was applied by spray-coat. As the three most efficient trialled detergents (OcGlu, MEGA-8, and DDM) appeared to have different specificities as to the mass range that they give rise to and enhance, one could think of mixing them to cover efficiently a more ample mass range. Data show that this is in fact a true prediction but there is still margin for further development in terms of improving the ion intensities. The hypothesis that protocols optimized for one type of samples may be directly transferred to another type of sample has not been entirely confirmed. Whereas, for example MEGA-8 has shown to generate a more efficient proteolysis at higher concentration both for fingermarks and rat brain tissue, the mixed detergent proteolytic solution is not equally efficient in the same ratios between the two different specimens and ad hoc method development is needed for every new tissue sample type. Finally, RapiGest SF that to date has only been reported for improved in-solution digestion has been successfully employed for on-tissue digestion (fingermarks and rat brain), and data suggest that further optimization of the concentration used is worthwhile. This study does show that proteolysis efficiency can be further improved, thus opening up the way to more reliable and efficient biomarker discovery and screening.

## Electronic supplementary material

Fig. S1 MALDI MS spectra of ungroomed fingermarks following proteolytic digestion performed by spraying the trypsin solution; the three detergents, OcGlu, DDM and MEGA-8, were tested at different concentrations of 0.5% (A-C), 1% (D-F) and 2% (G-I). (JPEG 704 kb)

Fig. S2 Peptide MALDI MS profiles displaying the mass region *m/z* 1360-3000 from rat brain digested with 20 μg/mL trypsin solution containing (A) OcGlu at 0.5% concentration and (B) MEGA-8 at 0.5%. (JPEG 99 kb)

Fig. S3 Peptide profile from the tryptic digestion of rat brain tissue with the mixed detergent composition at a ratio of 1:1:1 (OcGlu: DDM: MEGA-8); inset shows a zoom in the mass region 750-1790 Da. Tentative identification of MBP was made due to the detection of the peptides *m/z* 726 and 1339. (JPEG 259 kb)

Fig. S4 MALDI MS peptide profile of rat brain tissue digested *in situ* by spraying a trypsin solution containing 0.1% concentration RapiGest SFTM. The inset shows a zoom in the *m/z* region 800-1900. (JPEG 241 kb)
