# Supplementary figures and images for: Alternative Surfactants for Improved Efficiency of In Situ Tryptic Proteolysis of Fingermarks
Source: J Am Soc Mass Spectrom. 2015 Apr 28;26(6):862–72. doi: 10.1007/s13361-015-1140-z (PMC4422860; doi:10.1007/s13361-015-1140-z)

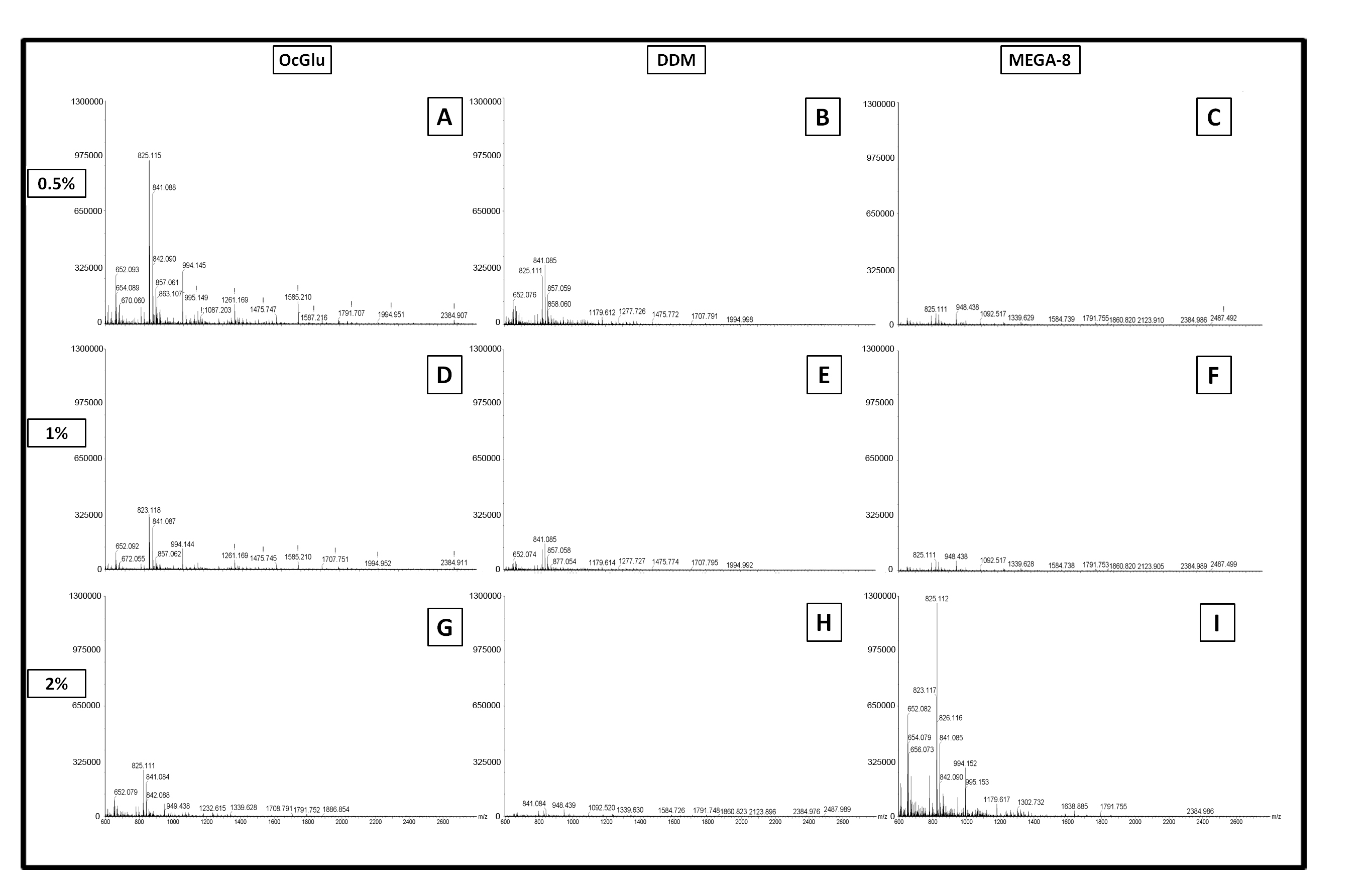

Supplement: Supplementary file 1 — MALDI MS spectra of ungroomed fingermarks following proteolytic digestion performed by spraying the trypsin solution; the three detergents, OcGlu, DDM and MEGA-8, were tested at different concentrations of 0.5% (A-C), 1% (D-F) and 2% (G-I). (JPEG 704 kb) [file 13361_2015_1140_Fig6_ESM.jpg]

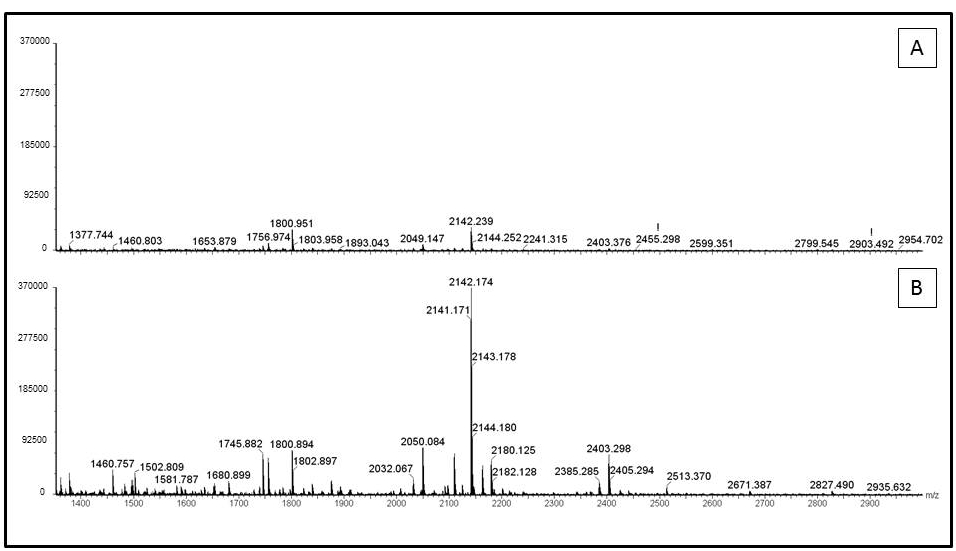

Supplement: Supplementary file 2 — Peptide MALDI MS profiles displaying the mass region m/z 1360-3000 from rat brain digested with 20 μg/mL trypsin solution containing (A) OcGlu at 0.5% concentration and (B) MEGA-8 at 0.5%. (JPEG 99 kb) [file 13361_2015_1140_Fig7_ESM.jpg]

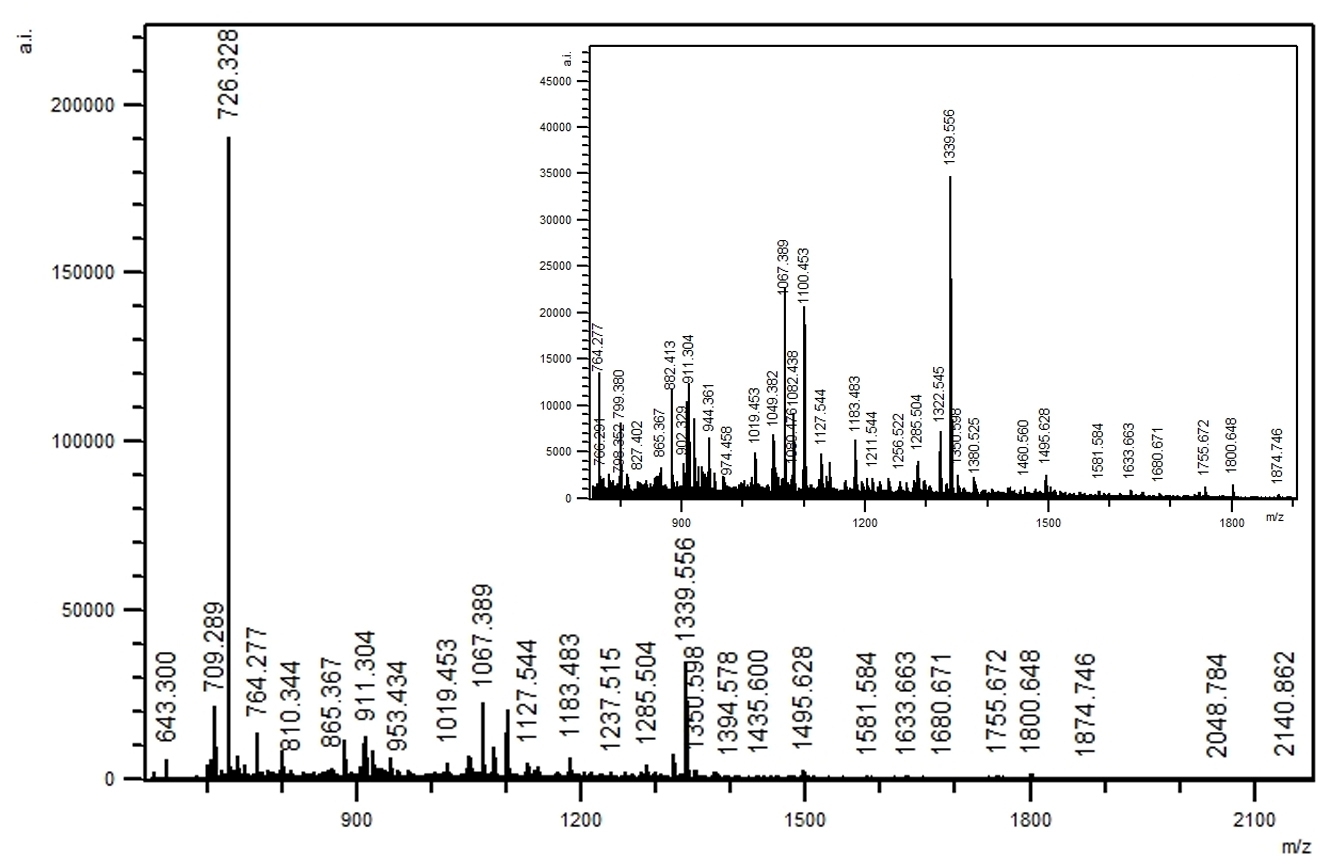

Supplement: Supplementary file 3 — Peptide profile from the tryptic digestion of rat brain tissue with the mixed detergent composition at a ratio of 1:1:1 (OcGlu: DDM: MEGA-8); inset shows a zoom in the mass region 750-1790 Da. Tentative identification of MBP was made due to the detection of the peptides m/z 726 and 1339. (JPEG 259 kb) [file 13361_2015_1140_Fig8_ESM.jpg]

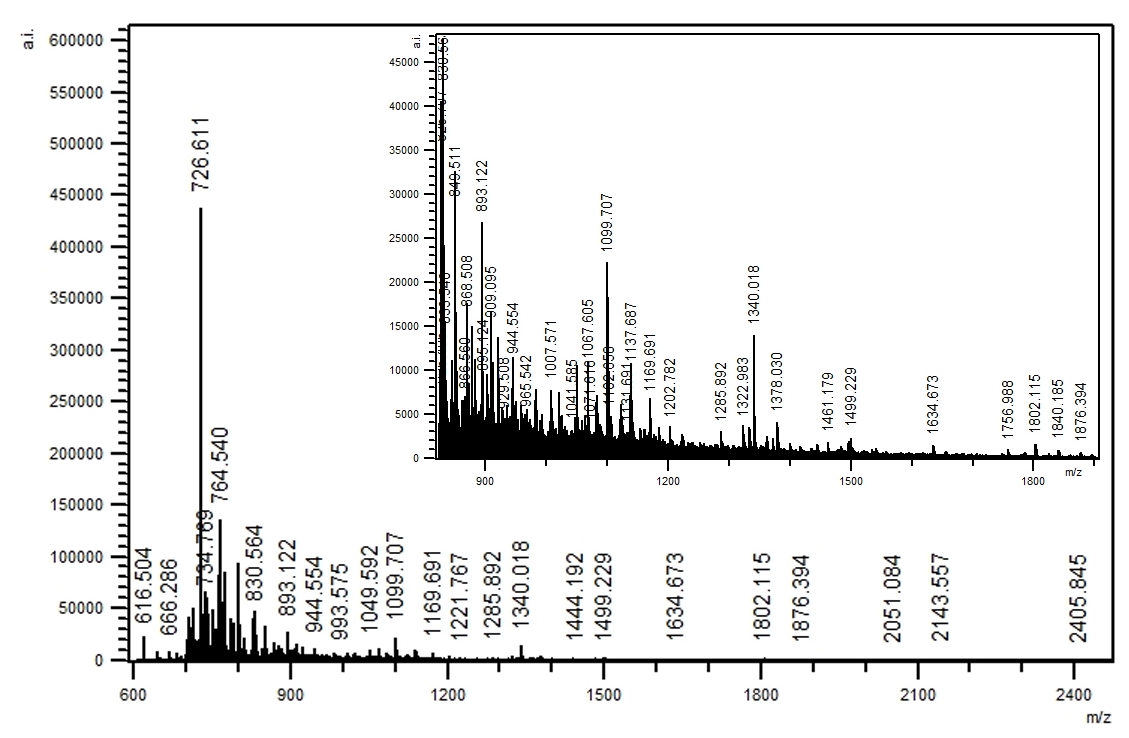

Supplement: Supplementary file 4 — MALDI MS peptide profile of rat brain tissue digested in situ by spraying a trypsin solution containing 0.1% concentration RapiGest SFTM. The inset shows a zoom in the m/z region 800-1900. (JPEG 241 kb) [file 13361_2015_1140_Fig9_ESM.jpg]
